# Different evolutionary pathways to generate plant fructan exohydrolases

**DOI:** 10.1093/jxb/erac305

**Published:** 2022-08-11

**Authors:** Wim Van den Ende

**Affiliations:** Laboratory of Molecular Plant Biology and KU Leuven Plant Institute, KU Leuven, Kasteelpark Arenberg 31, 3000 Leuven, Belgium

**Keywords:** Cell wall invertase, evolution, fructan, fructan exohydrolase, onion, vacuolar invertase

## Abstract

This article comments on:

**Oku S, Ueno K, Sawazaki Y, Maeda T, Jitsuyama Y, Suzuki T, Onodera S, Fujino K, Shimura H.** 2022. Functional characterization and vacuolar localization of fructan exohydrolase derived from onion (*Allium cepa*). Journal of Experimental Botany **73,**4908–4922.

**Fructans are fructose-based oligo- and polysaccharides serving as a carbohydrate reserve but also acting as prebiotics and potential signaling molecules. More than two decades ago, the scientific community first hypothesized that plant fructan exohydrolases (FEHs) could have evolved from vacuolar invertases, since plant fructans reside in the vacuole. However, it was found that FEHs resembled cell wall invertases instead. Oku *et al.* (2022) have now characterized a novel fructan 1-exohydrolase (1-FEH) from onion, an economically important species. This enzyme fits well within the vacuolar invertase subgroup, demonstrating that plant FEHs may be recruited from different types of ancestral invertases. This finding will inspire further research into the evolution of plant FEHs in general, shedding more light on their puzzling functions in both fructan-accumulating and non-fructan-accumulating plants.**

Plants use at least two but more often three to four different fructosyltransferases (FTs) to synthesize a diverse array of fructans, including inulins, levans, neo-inulin, and neo-levan types (with internal glucose residues, as occur in onion and ryegrass) and branched graminans (with 2,1 and 2,6 linkages, as occur in cereals) (Vijn and Smeekens, 1999; Van den Ende, 2013). For instance, inulin synthesis requires the action of 1-SST (sucrose:sucrose 1-fructosyltransferase) and 1-FFT (fructan:fructan 1-fructosyltransferase), while neo-inulin synthesis in onion requires 1-SST and 6G-FFT (fructan:fructan 6G-fructosyltransferase). It is not clear whether onion also harbors a genuine 1-FFT, as occurs in asparagus (Ueno *et al.*, 2020). To degrade more complex fructan profiles, a wider array of different FEHs is required. FEHs may act during reserve mobilization (in eudicots), but in monocots some FEHs can also act as ‘trimmers’ during fructan biosynthesis, limiting the degree of polymerization (DP) and branching (Van den Ende *et al.*, 2003). FEHs belong to the GH32 family. When they cut off terminal fructose moieties from inulin, levan, or graminan, they are termed fructan 1-exohydrolase (1-FEH), fructan 6-exohydrolase (6-FEH), and 6&1-FEH, respectively (Versluys *et al.*, 2022). Recently, a 6G&1-FEH, able to degrade neo-inulin-type fructans, was discovered in asparagus (Ueno *et al.*, 2018). 1-FEHs have so far only been described in fructan-accumulating plants, while 6-FEHs have been identified in several non-fructan plants where they may be involved in the degradation of fructans from levan-producing plant pathogens or rather act as fructan trimmers on levans originating from beneficial bacteria (Tian *et al.*, 2021; Versluys *et al.*, 2022). In the late 1990s, after the first plant FTs were cloned, the hunt was on to clone the first plant FEHs. While some groups selected cDNAs that resembled vacuolar invertases (VIs) for heterologous expression, other teams focused on the purification of native FEH enzymes, using peptide sequencing as the basis for FEH cloning. The latter approach proved successful (Van den Ende *et al*., 2000, 2001), surprisingly revealing that FEHs resembled cell wall invertases (CWIs) much more than VIs. In the last two decades, all characterized plant FEHs were found to fit well within the CWI/FEH subgroup. Oku *et al.* (2022) have now demonstrated that evolution followed a different path to generate a novel 1-FEH in onion with a vacuolar localization, fitting into the VI/FT subgroup instead.

## Onion: economic importance and physiological context

Onion (*Allium cepa*; Amaryllidaceae) is a major vegetable crop that is widely cultivated and therefore of huge economic importance (Box 1). The process of onion bulb initialization is accompanied by fructan accumulation (Oku *et al.*, 2019), similar to what is observed in developing barley kernels (Peukert *et al.*, 2014). Fructan pools are detected both in the leaves and in the bulbs. Oku *et al.* (2019) compared two different varieties showing different amounts of fructo-oligosaccharides (FOSs) in mature onions. During the first phase, a transient fructan peak was observed at the same moment and with the same magnitude in both varieties. This was also accompanied by increased 1-SST, 6G-FFT, and 1-FFT activities. Thereafter, fructans decreased as 1-FEH and invertase activities decreased, and the latter enzyme activities were higher in the variety that contained lower FOS levels at maturation. Also, during the last stages of onion bulb development, 1-FEH activities remained significantly higher in this variety, suggesting that a continuous trimming by 1-FEH inhibits overall FOS accumulation. Therefore, it was decided to search for the *1-FEH* gene and purify the native 1-FEH enzyme from developing onion bulbs (Oku *et al.*, 2022).

**Box 1. Onion: an economically important fructan accumulator widely used in food**

Onion is considered the third most important horticultural crop after potato and tomato. This healthy vegetable generates beneficial effects because it contains antioxidant, anti-inflammatory, immunostimulatory, and antiviral compounds (Peshev and Van den Ende, 2014). The abundant fructo-oligosaccharides (FOS) are believed to act as prebiotic compounds. Onion FOS consists of a mixture of low DP inulin- and neo-inulin-type fructans (Shiomi *et al.*, 2005). Both the basal stem, commonly known as the bulb, and the green parts are widely used as food ingredients.

## Evolution: different ways to create plant FEHs

**Fig. 1.**
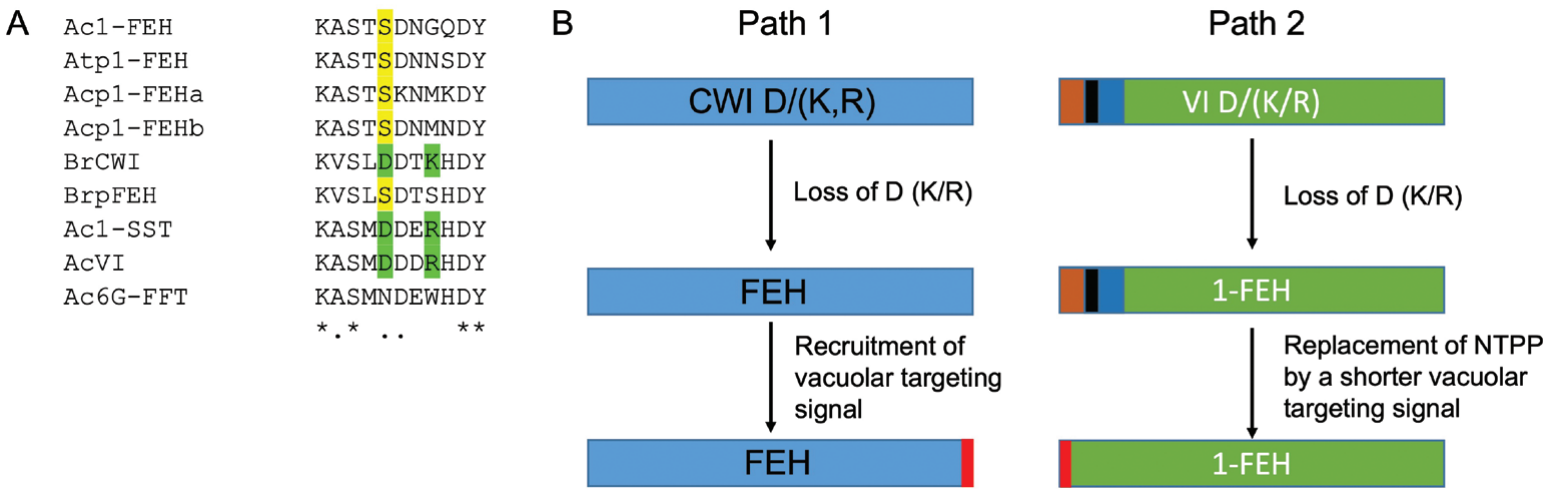
Two possible evolutionary paths to create plant FEHs, initiated by similar mutations in HVL1. (A) Multiple alignment of amino acid residues in HVL1 of selected plant GH32 members with known or predicted (p) functionalities. Residues in green highlight the presence of a D/R or D/K couple in S-type enzymes (CWI, VI, and 1-SST). A transition of D to S (yellow) is often observed regardless of whether a VI or a CWI was recruited to evolve the FEH. Ac, *Allium cepa*; At, *Agave tequilana*; Br, *Brassica rapa.* (B) The loss of the D/K or D/R motif is the proposed first step in the evolution of a plant FEH from CWI (blue, path 1) or VI (green, path 2). In path 1, the recruitment of a vacuolar targeting signal (red) is required to ascertain a vacuolar localization. In path 2, a complex N-terminal propeptide (NTPP) is already present for vacuolar localization. The NTPP consists of a central transmembrane domain (black) flanked by N- (brown) and C-terminal (blue) extensions. At a later stage, the NTPP can be replaced by a shorter vacuolar targeting signal (red).

To select the best candidate *1-FEH* genes for expression in *Pichia pastoris*, besides considering peptides from the purified native 1-FEH, the authors also considered previously established structure–function relationships within plant GH32 enzymes (Box 2). Prominent examples of the S-type to F-type transition can be found through the mutation of two specific amino acid residues in the KXSh**D**XX(**R/K**) motif in hypervariable loop 1 (HVL1). For instance, the transformation of a *Brassica rapa* CWI (BrCWI) into a putative BrFEH probably occurred through changing the D/K couple into S/S, while all surrounding amino acids remained unchanged (Fig. 1A). Strikingly, the D to S transition also occurred in the VI subgroup, comparing AcVI with Ac1-FEH (Fig. 1A), suggesting that similar mechanisms occur to develop 1-FEHs in both CWI and VI subgroups. As expected, onion S-type enzymes (VI and 1-SST) show the D/K or D/R couple while F-type enzymes (6G-FFT and the novel Ac1-FEH) do not. The recently released onion genome was searched to find other GH32 members that were not considered by the authors. Two additional genes with high identity to *Ac1-FEH* were detected, encoding putative 1-FEHs (termed Acp1-FEH1a and b) and showing amended HVL1 signatures (Fig. 1B). Interestingly, transcriptomics data suggest that *Acp1-FEH1a* and *b* are expressed in leaves while *Ac-1FEH* is mainly expressed in developing bulbs. Intriguingly, onion seems not to harbor two VIs as observed for most plant species (Van den Ende, 2013). Transcriptomic data confirm that there is only one fully active VI, although possible pseudogenes and partial VI sequences were detected in the genome. It seems that atypical VI-based 1-FEHs are not limited to the Amaryllidaceae since a VI-based 1-FEH sequence can also be found in *Agave tequilana* (Agavaceae; Cortes-Romero *et al.*, 2012; Fig. 1A) but not in *Asparagus officinalis* (Asparagaceae). Perhaps asparagus does not use a specific 1-FEH trimmer during fructan biosynthesis, fitting well with the fact that higher DP fructans are generated as compared with onion bulbs (Shiomi, 1993). Asparagus root fructans are degraded by a specific 6G&1-FEH and by VI during spear formation (Ueno *et al.*, 2018).

**Box 2. Structure–function relationships in plant GH32 enzymes**

It became possible to reasonably predict whether plant GH32 members belong to S-type (sucrose as donor: e.g. 1-SST and invertases) or F-type enzymes (fructan as donor: e.g. FEH, 1-FFT and 6G-FFT). S-type enzymes typically show a KXSh**D**XX(**R/K**) motif (h is a hydrophobic residue, X is any residue), while F-type enzymes lack a functional **D**/(**R/K)** couple to stabilize the glucose part of the sucrose donor. This couple is present in a hypervariable loop (termed HVL1) in the vicinity of the three catalytic residues at the very heart of the active site (Van den Ende *et al.*, 2009). The concept was confirmed through transforming a CWI (S-type) into a 1-FEH (F-type) (Le Roy *et al.*, 2007) and, the other way around, by changing a 6G-FFT (F-type) into an S-type 1-SST (Lasseur *et al.*, 2009).

From an evolutionary perspective, the recruitment of 1-FEHs from a VI (Fig. 1B, path 2), rather than from a CWI (Fig. 1B, path 1) has the benefit that VIs already have intracellular sorting mechanisms to transfer the enzyme to the vacuole, while CWIs have to recruit short N- or C-terminal vacuolar sorting motifs for that purpose (Van den Ende *et al*., 2000, 2001). On the other hand, maintaining the very long and complex N-terminal propeptide (NTPP; Xiang and Van den Ende, 2013), including a transmembrane region for membrane-based sorting, is rather costly. There may be an evolutionary pressure to replace this long motif by a shorter one. This may have occurred in Ac1-FEH where most of the NTPP sequence was lost but this did not (yet) occur in Acp1-FEH1a and Acp1-FEH1b, suggesting that these genes emerged later. An additional intriguing question is whether VIs can also be recruited to develop vacuolar 6-FEHs in levan-accumulating plants such as *Dactylis glomerata* or *Phleum pratensis*. In theory, an FEH may also evolve from an FFT by losing its affinity for fructan (and sucrose) as acceptor substrate.

## Conclusion and perspectives

The discovery that specific 1-FEHs can also be recruited from VIs opens many doors for further research in onion and related monocot species. It is striking that this specific evolutionary event only happened within the monocots. A survey of hundreds of eudicot VIs showed they all have the typical D/R or D/K couple in HVL1. Further structure–function and intensified phylogenetic research is required to better understand how widespread these VI-based 1-FEHs are within the monocots. Such insights may also clarify why eudicot FEHs seem to have been consistently recruited from CWIs. Importantly, the CWI/VI to 1-FEH transition not only requires the loss of the D/R or D/K couple in HVL1, it also requires modification of the substrate-binding site to allow binding of the larger fructan molecules. When specifically comparing sucrose (preferential substrate of CWI/VI) and 1-kestotriose (preferential substrate of Ac1-FEH), the loss of the D/R couple in Ac1-FEH prioritizes the binding of the subterminal fructose at the +1 subsite. However, an efficient binding site for the glucose part of 1-kestotriose at +2 had to evolve as well. It would be interesting to learn which amino acids in the active site of Ac1-FEH were specifically changed during evolution to stabilize glucose at the +2 subsite. Unravelling the 3D structure of Ac1-FEH and co-crystallization of 1-kestotriose and a mutated Ac1-FEH would serve the purpose.

Further research is required to clarify how the loss of the complex NTPP inherent to VIs was compensated by recruiting an alternative vacuolar targeting motif in Ac1-FEH, by revealing the identity of the motif and its precise location (either N- or C-terminal). Transcriptomics, qPCR, and the generation of CRISPR/Cas deletion mutants will be helpful to fully decipher the functional roles of Ac1-FEH, Acp1-FEHa, and Acp1-FEHb in onion, and their homologs in other monocot species. Finally, it would be interesting to further explore whether the functional shift from a VI to a 1-FEH is associated with a regulatory shift at the level of the *1-FEH* gene promotors.
